# The Influence of Human–Animal Interactions on Mental and Physical Health during the First COVID-19 Lockdown Phase in the U.K.: A Qualitative Exploration

**DOI:** 10.3390/ijerph18030976

**Published:** 2021-01-22

**Authors:** Emily Shoesmith, Lion Shahab, Dimitra Kale, Daniel S. Mills, Catherine Reeve, Paul Toner, Luciana Santos de Assis, Elena Ratschen

**Affiliations:** 1 Department of Health Sciences, University of York, York YO10 5DD, UK; elena.ratschen@york.ac.uk; 2 Department of Behavioural Science and Health, University College London, London WC1E 7HB, UK; lion.shahab@ucl.ac.uk (L.S.); dimitra.kale.09@ucl.ac.uk (D.K.); 3 School of Life Sciences, University of Lincoln, Lincoln LN6 7DL, UK; dmills@lincoln.ac.uk (D.S.M.); lassis@lincoln.ac.uk (L.S.d.A.); 4 School of Psychology, Queen’s University Belfast, Belfast BT9 5BN, UK; c.reeve@qub.ac.uk (C.R.); p.toner@qub.ac.uk (P.T.)

**Keywords:** human–animal interaction, human–animal relationships, companion animals, non-companion animals, COVID-19, mental health, wellbeing, loneliness, social isolation, lockdown

## Abstract

The coronavirus disease 2019 (COVID-19) pandemic presents an opportunity to explore the role of animals as sources of emotional and physical support during a period when most of the population is experiencing social and environmental challenges. We investigated how companion animal owners perceived the influence of human–animal interaction on their physical and mental health during the first COVID-19 lockdown phase in the U.K., and what concerns they had regarding their animals at this time. We also explored the impact of participants’ interaction with non-companion animals during this phase. A cross-sectional online survey of U.K. residents aged over 18 was conducted between April and June 2020. The final item of the survey invited open-ended free-text responses, allowing participants to describe any experiences and/or perceptions of their human–animal relationships during the COVID-19 lockdown phase. A qualitative thematic analysis of responses was undertaken. Four main themes related to the following aspects of human–animal interactions during the COVID-19 lockdown phase were identified: the positive impact of animal ownership during the COVID-19 lockdown (e.g., amelioration of wellbeing and mental health), concerns relating to animal ownership during the COVID-19 lockdown (e.g., concerns over animals carrying the COVID-19 virus), grief and loss of an animal during the COVID-19 lockdown and the impact of engaging with non-companion animals during the COVID-19 lockdown. The findings complement and extend previous insights into the impact of human–animal interaction with both companion and non-companion animals. They also highlight the challenges of caring for an animal during the lockdown phase and indicate the need to consider the development of further targeted support strategies, such as “day care” for the companion animals of key workers in this context.

## 1. Introduction

The concept of the human–animal bond is well-established [1,2], where it is generally acknowledged that relationships between humans and companion animals can be enduring and meaningful [1]. The belief that “pets are good for human health” [3] is gaining increasing popularity despite the acknowledgement that the research evidence in this area offers contradictory findings and often encompasses substantial methodological weaknesses [4,5]. For example, studies have reported that animal ownership has benefits for the individual, including enhanced mental health outcomes, such as decreased anxiety [6], stress reduction, improved quality of life, enhanced social and community interaction [1,7,8] and coping with challenging situations [9,10]. By contrast, multiple studies have also found negative effects of animal ownership, including a higher likelihood of experiencing depression [11] and a decline in mental health [12,13,14]. It has been suggested that animals may exacerbate depressive symptoms due to various responsibilities and emotional attachments related to animal ownership [13,14]. There is a consensus in the field that further research investigating the links between human–animal relationships and mental health and wellbeing is required [15].

Over 40% of United Kingdom households are estimated to own at least one companion animal [16]. The COVID-19 pandemic presents an opportunity to explore the role of companion animals as sources of emotional and physical support during a period when the vast majority of the population is experiencing social and environmental challenges [9,10]. Previous studies have reported that animal ownership appeared to mitigate some of the detrimental psychological effects of the COVID-19 lockdown phase in the U.K. [9] and Spain [10]. However, there were also numerous stressors related to animal ownership, such as behavioural, welfare, financial and logistical concerns, which are all likely to be amplified by the pandemic [9,10,17].

While recent COVID-19-related quantitative studies found a positive association between animal ownership and mental health outcomes [9,10,18], a better understanding is needed to explore how animals influence human’s mental and physical health. Therefore, the overall aim of the current study was to explore the impact of the first COVID-19 lockdown phase in the U.K. on animal owners, their animals and the relationships between them. Specifically, we aimed to understand how companion animal owners perceived human–animal interaction as influencing their physical and mental health during this phase and to understand any concerns that animal owners may have had regarding their animal at this time. Furthermore, we were also interested in exploring the impact that interaction with non-companion animals (e.g., wildlife) had on participants during the lockdown phase.

## 2. Materials and Methods

### 2.1. Study Design

A cross-sectional study with free-text responses to an open-ended item.

### 2.2. Settings and Participants

The survey was conducted by questioning the U.K. general population. All U.K. residents over 18 years of age were eligible to take part, irrespective of companion animal ownership.

### 2.3. Measures

As described in detail elsewhere [9], a bespoke questionnaire was developed by a multi-disciplinary team of academics with input from third sector animal welfare and training organisations. The survey included the option for participants to leave an open-ended, free-text comment to describe their experiences and perceptions of their human–animal relationships and interactions during the COVID-19 lockdown phase in the U.K. The item read: “If you have any further comments related to your experience with animals during COVID-19, you can leave them here”.

### 2.4. Recruitment and Procedures

The survey was released in the Qualtrics survey software (Qualtrics, Provo, UT, USA) and promoted using academic and third sector networks (including animal charities with an interest in human–animal interaction research), social media (Facebook, Twitter) and other media outlets (e.g., Reddit). The study commenced on 16 April 2020, four weeks after the first strict social distancing and social isolation measures came into force in the U.K., and ended on 14 June 2020, when the first lockdown measures were officially eased. Prospective participants followed a link to the survey, where they were presented with a participant information sheet and consent form. Consent to participate in the anonymous survey was indicated by ticking an online check box. A screening question requiring participants to name their country of residence denied access to non-U.K. residents. All data were stored on the secure Qualtrics server at the University of York.

Ethical approval for the survey was granted by the Health Sciences Research Ethics Committee at the University of York, U.K., on 16 April 2020.

### 2.5. Data Analysis

Responses to the free-text question were exported to NVivo 12 software (QSR International Pty Ltd. Version 12, Ottawa, ON, Canada). The free-text comments were analysed using thematic analysis [19], employing an inductive approach, in which coding and theme development were driven by the content of the responses. One author (E.S.) familiarised herself with the data by reading all responses and notes were made of any potential codes by identifying recurring words or units of meaning. Subsequently, the same author generated initial codes from the data and organised them into meaningful groups. Codes were then organised into potential themes and all relevant coded responses were collated within the identified themes. Two authors (ES and DK) independently reviewed the construction of themes and relevant quotations to agree to the assignment of themes.

## 3. Results

A total of 5926 participants consented and were eligible to take part in the study. Of those, 934 provided a response to the optional free-text item (see Table 1 for participant characteristics of this subsample). Full participant characteristics for the whole survey sample are provided elsewhere [9].

The thematic analysis of free-text responses, many of which included substantial detail and were characterised by narrative depth, resulted in the identification of four main themes with associated clusters of sub-themes related to various aspects of human–animal interaction during the COVID-19 lockdown phase (see Table 2). To illustrate themes and sub-themes, the free-text responses are presented as verbatim quotes below, with the gender and age range of participants provided in brackets.

### 3.1. Positive Impact of Animal Ownership during the COVID-19 Lockdown

#### 3.1.1. Amelioration of Wellbeing and Mental Health

There was a consensus that companion animals constituted a reliable source of support, providing unconditional love, affection and companionship that fostered relationships that were free from judgement and conflict. Companion animals were frequently perceived as being able to enhance mood, reduce stress, and help participants to cope generally with the COVID-19 lockdown phase, for example, by offering a “*constant non-judgemental source of affection*” (female, 35–44). Participants frequently described positive experiences of how their animals had influenced their current life situation and associated mood states. For example, many participants referred to depression and/or anxiety diagnoses and described how their animal had provided a constant source of companionship and emotional support.

Animals are vitally important for keeping my anxiety and depression from spiralling especially in times like these and being in isolation.

(female, 18–24)

My rabbit really boosts my mood and eases my anxiety. She gives me purpose in such uneasy times.

(female, 18–24)

*My Westie* [West Highland White Terrier dog] *is a huge support. I suffer anxiety and mild agoraphobia […] without my dog/best friend, I would not leave the house despite living on a quiet farm. She keeps my anxiety levels down by talking to her when I’m out and breaking my focus on anything negative. I am terrified of catching COVID-19 but having* [dog’s name] *keeps me mentally and physically better in the current environment.*

(male, 35–44)

I cry two or three times each day as bad COVID news comes in and I worry about my two daughters on the frontline. But I know this is a normal way of coping with stress. I cried rarely before COVID. When I cry, I often talk to my dog and stroke him.

(male, 65–70)

One participant described how different animal species provided emotional support for her in different ways:

All my animals have helped keep me motivated, calm and happy during this time, not just the one I am closest to. Different species have different qualities: dog is close companion, but reptiles are interesting and calming to watch, guinea pigs are entertaining, general pet care tasks give purpose and structure to my day.

(female, 25–34)

Some participants reported that their animals were able to provide unique emotional support as a result of their ability to respond to their owners in an intuitive manner, especially in times of distress.

*My whippet has really picked up on this, he stays by my side constantly and feeds and mirrors my emotions* […] *he is hyper vigilant to my needs giving lots of attention and affection accompanying me to the kitchen or bathroom, to hang washing out.*

(female, 35–44)

My dog is very perceptive and knows if I am feeling worried or concerned and this has helped a lot, now and in the past.

(female, 65–70)

Additionally, it was clear that animal ownership gave participants a sense of purpose and focus during the COVID-19 lockdown phase, promoting routine and consistency to daily life, which had inevitably changed for many. The responsibilities of animal ownership were frequently perceived as offering a distraction and were thus perceived as positively influencing participants’ mental health and wellbeing. Animals also appeared to be a source of motivation to engage owners in activities, and participants often described how their animals played an important role in enabling them to have something productive to look forward to.

My dogs give my life structure and routine, which is very important for my mental wellbeing and peace of mind.

(female, 55–64)

Me and my fiancé have two guinea pigs and a hamster, and they have made our experience of lockdown so much better. It gives me something to look forward to every morning and a bit of routine and consistency to my life which has otherwise completely changed.

(male, 18–24)

My horse has also given me just as much companionship, happiness and positivity during the time. I spent 2–3 h caring for her every day and I don’t know how I would be coping if I couldn’t do that. She has been my wings and my hope during these difficult times.

(female, 25–34)

#### 3.1.2. Reduction of Loneliness and the Facilitation of Social Support

The constant source of companionship appeared to ameliorate feelings of loneliness. This was particularly the case for those living alone, or those who lived with key workers who were frequently working outside of the home.

My dog is helping me cope and making me feel less alone during this, since I’m not getting much communication from my friends (who I don’t live with)–you are never short of a friend when you have a dog! She is a constant source of joy and brings great comfort in tough times like these.

(female, 18–24)

I don’t know how I would have coped without her, as I am alone all day with my partner being a key worker still working outside of the home during the pandemic.

(female, 18–24)

Physical contact with animals was also described by some as helping to reduce feelings of loneliness and isolation while living alone.

As a single person, I’ve missed hugs from my friends and family. Having physical contact with my dog lying next to me, or stroking him, grooming him has had extra value.

(female, 55–64)

Animals also appeared to offer a distraction when human social interaction was dominated by discussions regarding the pandemic, which could negatively impact an individual’s mental health and wellbeing. Therefore, verbally interacting with a companion animal may have provided a sense of “connectedness” or normalcy.

My pet hamster has been my main emotional outlet during COVID-19–I find myself talking to her. Friends and family are all worried about the virus and that takes up most of their communication (which I don’t find good for my mental health), so I end up talking to my hamster about everyday things–what I’m having for dinner, TV, how work is going. I suspect this is exacerbated by living alone! She’s also a comfort in having a companion in my tiny flat–there’s another ‘presence’ here.

(female, 25–34)

However, a number of participants stated that despite the strong bond with their animal, this did not replace the yearning for human social contact that had been considerably restricted during the COVID-19 lockdown phase.

My dogs and cats are not a replacement for human contact, which I am sorely missing. But they have been a welcome distraction and have given me something to focus on and stopped me from feeling so lonely.

(female, 25–34)

Despite this, many participants stated that animal ownership resulted in engagement in animal-related activities, which subsequently led to increased socialisation with friends or family. In this sense, their animal was perceived as a connection within their social network, which also provided a sense of belonging in their respective communities.

My dog has definitely made lockdown bearable. He is great company, a constant companion and living creature with which to share my day and has helped me to get to know my neighbours. I have made a few really good friends through our daily walks in the park.

(female, 55–64)

She is constantly happy, has her routine which forces us to keep a schedule and she brings our family together.

(female, 35–44)

#### 3.1.3. Encouragement of Physical Activity

Many participants commented that owning an animal encouraged and promoted physical activity. Animals appeared to enhance mobility, increase participation in exercise and promote contact with nature, especially for owners of dogs and horses.

I am lucky enough to own horses that I keep on my own land, so all my exercise is taken there when I go to check and feed my horses every day.

(female, 70+)

I think having a dog and living on my own has made me have structure to my day, spending a good few hours at a time out of the house every day in the local countryside/moors walking for the one permitted period of exercise. I wouldn’t do this without my dog, and certainly not spend as long outside as I do.

(female, 55–64)

I am very anxious about leaving the house for exercise since COVID-19 as I’m worried about other people’s reactions. My dogs mean I go out every day for exercise. Without them, I don’t think I’d go out at all during this time.

(female, 25–34)

*I have mobility problems due to advanced spinal deterioration.* [My dog] *keeps me active by going for walks.*

(male, 35–44)

#### 3.1.4. Increased Appreciation of Animals during the COVID-19 Lockdown

Participants frequently mentioned that they appreciated their animals and the emotional support they provided even more during the pandemic than they had done before.

Appreciate the love and loyalty of my two dogs more than ever!

(female, 70+)

*My animals* [dogs and cats] *have always been extremely important to me; they are my family. During this time, they are my life.*

(female, 70+)

The notion that individuals “*could not live without*” their animals during this time and that they were a “*godsend*” or a “*lifeline*” in the pandemic was also frequently expressed.

I couldn’t have got through this without my dog.

(female, 35–44)

Feel beyond blessed and fortunate to have a dog. I am closer than ever with him. He is my entire world and life will not be worth living without him.

(female, 45–54)

### 3.2. Negative Impact and Concerns of Animal Ownership during the COVID-19 Lockdown

In addition to the positive benefits reported by companion animal owners, negative aspects to animal ownership during lockdown were described. Owners reported they were often worried or concerned about various elements of ownership, including the possibility of animals carrying the COVID-19-causing virus, access to veterinary care, caring for their animals, and concerns about their animal experiencing separation-related problems upon their return to work. It was apparent that these concerns often exacerbated stress in participants due to the responsibilities and potential additional financial cost of pet ownership.

#### 3.2.1. Concerns over Animals Carrying the COVID-19-Causing Virus

Many participants expressed concerns about their animals carrying the virus in their fur, and the possibility of this being transferred to the owner via physical contact with the animal (e.g., grooming, playing or petting the animal). One participant highlighted that their mental health had declined as a result, as she was anxious about petting her cats due to the possibility of transmission.

I was enjoying the time I spent with my cats a great deal who seemed more affectionate until I read on the RSCPA website that if your cat goes outside, you should limit contact with them. Now I’m afraid of stroking my cats and that has impacted on my mental health. I’ve felt this as a huge loss.

(female, 55–64)

I felt anxious about the uncertainty as to whether or not the virus can be carried on dogs’ coats, and the measures I should take to mitigate the associated risks.

(female, 35–44

However, a number of participants expressed frustration at this perception and urged other owners not to relinquish their animals in response to the possibility of transmission of COVID-19 from animal to owner.

People should NOT dump their animals in case they can catch COVID-19, my cat means the world to me. I would never get rid of her.

(female, 55–64)

I have been concerned by reports in the media implying that pets can spread COVID-19. It worries me that others might abandon or get rid of their pets as a result.

(female, 55–64)

#### 3.2.2. Animals’ Potential Separation-Related Problems and Returning to Work after the COVID-19 Lockdown

One of the most frequently cited concerns about animal ownership was the possibility of animals developing separation-related problems once the owner returned to work after an extended period at home. Many participants expressed that they had “*felt closer*” to their animals since the lockdown phase began and often reported that their animal had been “*clingy*” or “*needy*”. It was apparent that the possibility of separation-related problems was adding additional stress to the owner, as participants frequently mentioned they were worried about returning to work due to the impact this would have on their companion animal.

*My main concern is he* [dog] *is even more attached to me than he was before and often lies staring at me, so I do really worry what it will be like when I return to work.*

(female, 45–54)

I have a rescued cat which needs a lot of company—I am very concerned about his anxiety levels/separation issues once I return to work.

(female, 65–70)

My dog has become a lot more needy and howls if I leave the house without him, even if it’s just to do some gardening and he can’t see me. Going back to work will be very hard on him.

(female, 45–54)

#### 3.2.3. General Animal Welfare Worries

A number of participants expressed their concerns about their animals’ health and wellbeing during the COVID-19 lockdown phase. This was often exacerbated by the possibility that their access to veterinary care would be restricted, as appointments were often limited to emergency care only. Those requiring routine appointments (e.g., vaccinations, flea treatment) were often delayed, which resulted in additional stress for the owner.

*One of my pets* [cat] *injured herself badly the past couple of weeks. It’s been quite stressful to do telephone consultations, pick up prescriptions at the door of the vet (can’t go inside), decide whether I should argue she’s an emergency case and should be seen in person without really being able to talk to the vet and explain everything properly.*

(female, 18–24)

*We also* [our cats] *have fleas and feel unable to go to the vets at this time for advice.*

(female, 25–34)

Concerned with the level of support provided by vets since lockdown. Inoculations unlikely to be given.

(male, 55–64)

Arranging alternative care for an animal whilst the owner was working outside of the home was often cited as a concern of animal ownership. Many participants, primarily dog owners, noted that animal care that was readily available prior to lockdown had since been restricted, and this had resulted in feelings of anxiety. Some participants suggested that it would be beneficial for animal care to be easily accessible for key workers (e.g., healthcare professionals) in the context of increased working hours.

COVID-19 has been difficult for me and my dog, especially sorting out care for him while I am at work.

(female, 25–34)

I’ve had to stop working mainly from home and have gone back to work in the hospital. This has caused some anxiety about finding ways to make sure our dogs do not spend too long by themselves. Doggy day care for keyworkers would have been a good idea!

(female, 35–44)

#### 3.2.4. Financial Difficulties and Concerns

COVID-19-related financial concerns that may impact the owner’s ability to care for their animal were mentioned by some participants. Due to financial uncertainty, participants expressed their concern over buying pet food and other necessities, being able to provide healthcare if required and maintaining their animal’s insurance.

*I worry for their* [dog and cat] *health and wellbeing during these times, with the concern over financial difficulties if work becomes scarcer. They are important members of our family.*

(female, 55–64)

*My only concern is I haven’t been able to keep them all* [dogs and cats] *insured and that’s a worry.*

(female, 35–44)

### 3.3. Grief and Loss of an Animal during the COVID-19 Lockdown

The grieving process of losing an animal was often felt to be exacerbated by the restrictions in place due to the COVID-19 lockdown.

Suffered the loss of a much-loved dog, made worse by confinement and vet restrictions. Too much time indoors to distract yourself so grieving was and is more intense.

(male, 65–70)

*We got online vet advice and drove further than usual to a vet where they could see her and put her* [cat] *to sleep. This bereavement was hard anyway but extra hard due to the sad and anxious times.*

(female, 45–54)

Some participants had a positive experience with their veterinarian at this time, despite the lockdown restrictions in place.

*My vet was wonderful and gave me a mask so I could be there at the end* [with my cat]. *This was so helpful to me in dealing with the grief.*

(female, 55–64)

A number of participants expressed feelings of guilt during their grieving period if they had been working additional hours due to the pandemic. As a result, participants highlighted they had been spending less time with their animals.

I lost one of my pet rats during the COVID-19 situation and I found it difficult as I was unable to give him a proper goodbye. I felt guilty as I hadn’t been spending time with him recently due to working increased hours.

(Other, 18–24)

However, a number of participants expressed that their other animals had facilitated their ability to cope throughout the grieving process and were often referred to as “*my boys*”, “*my girls*”, “*my furbabies*”, “*my furkids*” or “*my children*”. These anthropomorphisms clearly illustrate the intensity of the human–animal bond and how animals are a strong source of emotional support during these challenging times.

*My dog passed away within a few days of the lockdown being in place … I have had to deal with that and mourn her without any physical support from anyone apart from my two boys* [my cats].

(female, 45–54)

I had four dogs and sadly lost my very elderly German shepherd in week 1 of lockdown. I honestly do not know how I would have coped had I not had my other girls to take care of. We have four grown up children, but these girls are like my children. They somehow make this world a better pace to be, for me anyway.

(female, 45–54)

### 3.4. Positive Impact of Interaction with Non-companion Animals during the COVID-19 Lockdown

There was a general sense that interactions with non-companion animals (e.g., wildlife) and frequent contact with nature had a positive impact on mental health. Participants often reported a sense of awe and privilege when seeing animals within nature. Some participants suggested that seeing an animal in their natural environment provided opportunities for distraction from their inner feelings of distress due to the pandemic.

*We’ve gone out to see which birds are on the local flooded fields and submit the data to BirdTrack. We’ve always felt better afterwards* […], *the birds, and my wife have helped me to control my anxieties about the risks of COVID-19 to my loved ones and me, and fears about losing my job.*

(male, 55–64)

Wildlife in the garden (which includes birds, bats, foxes and squirrels) have been a great joy and comfort during the long days.

(female, 55–64)

A number of participants noted that interactions with non-companion animals provided a source of companionship and ameliorated feelings of loneliness.

I go and feed the geese at the campus, that’s very comforting, makes me feel less alone. They sometimes run to me and I feel like someone is waiting for me.

(female, 18–24)

Some participants described how spending time at home watching garden birds had resulted in a sense of familiarity with and knowing the birds.

Staying at home has given me the opportunity to really watch the blackbirds and robin take mealworms back to their nests. I feel I know them! There’s a pheasant nesting under my hedge and I hope to see the hen lead the chicks away from the nest when hatched.

(female, 65–70)

Always enjoyed watching birds in my garden but now they are main focus of the day–watching them at bird feeders and watching them gather material for nests and their antics in general. Spent more time sanitising bird feeders and putting nesting material out for them. Feel that they are my friends now and the experience is helping me get through this time.

(female, 55–64)

When participants saw non-companion animals in nature, this often motivated participants to engage in other animal-focused activities. For example, one participant noted that they “*observe birds in the garden and then read up on their behaviours*”. Another example included bird watching leading to “*unexpected exchanges with birding friends and members of a local birdwatching Facebook group*”. Lastly, a number of participants stated that they had found other animals situated in proximity to their homes and had been unaware of these until the lockdown phase was implemented. Discovering these animals had led participants to visit them more frequently as part of their routine.

We’ve discovered alpacas five minutes from our house that we never knew were there. We now go and see them several times a week.

(female, 45–54)

We have been walking more and found a local pond with lots of birds and have been visiting several times per week since lockdown.

(female, 25–34)

Myself and my partner have been seeking out animals on our daily walks every day. We were always visiting nature reserves and have now found some good places to birdwatch within a four-mile radius of our home. We have found a heron, and have been watching the chicks grow up, along with many ponds with various ducks and geese.

(female, 25–34)

## 4. Discussion

The findings from this qualitative study not only support notions of the benefits of human–animal interactions with both companion and non-companion animals but also highlight the challenges of caring for an animal during the lockdown phase, for example, concerns over animals carrying the COVID-19 virus, potential changes in attachment and future separation-related problems when the owner returned to work, along with other animal welfare issues, such as restrictions to veterinary care and the ability to provide adequate care for animals. Overall, the results indicated that animal ownership had a positive impact on mental and physical health during the COVID-19 lockdown phase in the U.K., as purported by various mechanisms, including the promotion of health through companionship and emotional support and encouraging physical activity, thus offering a distraction from inner feelings of distress, providing a source of motivation to engage owners in activities, responding to owners in an intuitive manner and providing a sense of connectedness or “*normalcy*”; however, there were also significant concerns that might have outweighed these in some cases.

### 4.1. The Relationship between Animal Ownership and Health Benefits: Potential Mechanisms of Effect

The findings from this study support and extend insights related to potential mechanisms of benefits that were previously identified. According to existing evidence, animal ownership may encourage physical activity [20], which is a mechanism that is strongly supported in the case of dog and/or horse owners. Previous literature has suggested that owning a dog increases levels of physical activity [21,22] and can have subsequent health benefits [23]. Likewise, high physical activity has been reported by horse owners [24]. Existing research has indicated that dog and/or horse ownership was one of the strongest factors for participation in outdoor physical activity [25]. This aligns with the findings in the current study, as many of the responses related to the promotion of physical activity were primarily from dog and/or horse owners. Higher levels of physical activity are associated with improved health [26] and quality of life [27], suggesting owning animals that encourage physical activity may have an impact on owners’ mental health and wellbeing.

Previous research has hypothesised the mechanisms through which animals may affect mental health and wellbeing, and leading theories involve the effects of social support. For example, the “buffering” hypothesis suggests that social support effects are notable only in the presence of stressors [4]. One study examined whether animals could moderate the anxiety-inducing effects of a stressful situation [28] and found that petting a real animal significantly reduced anxiety. This suggests the interaction with and feedback from the animal may play an important role in emotional regulation. This aligns with the current findings, as we found that animals were able to provide unique emotional support as a result of their ability to respond to their owners in an intuitive manner, especially in times of distress.

However, the current findings also extend our understanding of potential mechanisms of benefits that were previously identified in dogs to other species [29]. Our results indicate that the benefits derived from animal ownership (including a variety of species) or even interaction with animals in the wild may arise by offering a distraction from inner feelings of distress, providing a source of motivation and a sense of connectedness or normalcy. Previous qualitative evidence has identified feelings of normalcy and positive distraction as a possible mechanism for the benefits of human–animal interactions; however, this was focused on dogs [29] or animal-assisted interventions [30] rather than animal ownership in general. For example, research on animal-assisted interventions has suggested that a positive distraction is a major benefit and is more than just emotional support and companionship, as animals can distract from pain, stress and other difficulties [31,32,33], as well as foster feelings of normalcy [32,33]. It has recently been suggested that dog–human-related activities might be linked to specific hedonic wellbeing, life satisfaction and eudemonic wellbeing outcomes, including those described here [29]. Thus, our findings not only provide further validation of the framework proposed by these authors for understanding how activities associated with dog ownership might relate to human well-being [29] but also extend these mechanisms of benefit to other species and to human–animal interactions with both companion animals and non-companion animals within nature.

### 4.2. The Challenges of Caring for Companion Animals during Lockdown

It was evident that the vast majority of animal owners in this study perceived their animals as helping them cope with the first COVID-19 lockdown phase, offering an important source of emotional support. However, concerns and worries relating to caring for their animals during this time were frequently reported and were likely to have exacerbated feelings of stress for the owner. It was apparent that there was a range of COVID-19-specific issues, including animal care for key workers. Interestingly, a number of participants (primarily dog owners) suggested that it would be beneficial for animal care to be easily accessible for key workers, as the ready availability of animal care prior to the lockdown phase had been restricted and many were working additional hours due to the pandemic. This resulted in participants feeling particularly anxious about leaving their animal(s) alone for extended periods and not being able to provide adequate care (e.g., restricted amount of dog walking). Some participants’ suggestions of organising day care for the dogs of key workers in this context could be further explored.

Many concerns expressed in our study are similar to those found in previous research investigating the human–animal bond and the confinement period, as animal owners reported concerns about their animal’s health, obtaining animal food and restricted veterinary access, and worried the animal would not adapt well post-lockdown [10]. These concerns could be an indication of an underlying state of worry or anxiety as a result of the COVID-19 pandemic. For example, if a participant is experiencing greater anxiety due to the current circumstances, they may project more anxiety onto their animal. Previous literature has reported that highly anxious individuals are more likely to report a greater level of concern about their animals [34]. This seems plausible and should be further investigated, given that the COVID-19 outbreak has resulted in large levels of uncertainty, and fear of the unknown is a fundamental component of anxiety-related disorders [35].

Alongside the concerns frequently listed, the loss of and grief for an animal was clearly exacerbated during the COVID-19 lockdown phase. Owners described that dealing with their loss was more difficult, as the confinement period restricted opportunities for the participants to distract themselves. Previous research consistently supports the notion that those experiencing the grieving process of a companion animal may often perceive a lack of support from their social network and the professional community, as the human–animal relationship can often be misunderstood and undervalued [36,37]. Therefore, this may increase the risk of further social isolation during the grieving process [38], which is already amplified due to the COVID-19 lockdown phase. This risk will inevitably become greater when the person had previously relied on their companion animal for emotional support. The findings from this study suggest that other companion animals offered a strong source of support for owners during the grieving process, with a number of participants highlighting that they would not have been able to cope without their presence.

## 5. Limitations

We acknowledge a number of limitations to the current study. First, the subsample of participants who provided a free-text response was predominantly female, which is a bias that is common in the field of human–animal interaction research [39] and has been found in a number of studies of animal ownership when recruitment was voluntary [10,40,41]. Although gender differences have been identified for some elements relating to human–animal interaction [42], this does not appear to be the case for certain aspects connected to the intimacy domain of the human–animal bond [43]. Therefore, our predominantly female sample may not have affected our results substantially.

The participants were also predominantly below 65 years, resulting in a lack of representation of respondents in the 65+ age band, which is a group that is known to be susceptible to social isolation and depression [18,44]. Moreover, the subsample consisted largely of animal owners, but this is not surprising given the whole survey sample included only 603 (10.2%) non-animal owners [9]. Participants were not required to indicate the length of time they had owned their companion animal. Given the large increase in people adopting or buying companion animals during the COVID-19 pandemic and subsequent consideration of relinquishment due to economic hardship or care routine difficulties [45], there may have been differences in reported concerns between the established and non-established owners. However, as described elsewhere [9], nearly all participants (99.7%) reported that they had not considered giving up their animal(s) since the start of the pandemic. A further limitation is that we did not collect data related to household income. As COVID-19-related financial concerns were mentioned by companion animal owners, this would differentially affect various income groups and would be important to explore in future research.

Lastly, we were not able to elicit themes and sub-themes further as we did not interview or otherwise directly interact with the participants. Rather, the responses were obtained from a free-text response survey item. Therefore, it is unclear whether there is data saturation in the same way that could have been achieved in face-to-face interviews with a theory-based interview schedule. As we were unable to prompt participants to elaborate or provide further information, the richness of the data was limited compared with semi-structured interviews.

## 6. Conclusions

In conclusion, our study provided in-depth insight into the impact of human–animal relationships and interaction with both companion and non-companion animals in a COVID-19 lockdown context. It highlighted the role of animals as sources of emotional and physical support during a period when most of the population is experiencing social and environmental challenges, supporting and extending insights into potential mechanisms of benefits previously identified. However, the study also highlighted specific challenges that are associated with caring for a companion animal during the lockdown phase, which can often exacerbate feelings of distress for the owner, indicating areas such as access to veterinary care, dealing with the loss of an animal and arranging day care for key workers’ dogs, in which future support strategies could be developed.

## Figures and Tables

**Table 1:** Participant characteristics (*n* = 934).

Characteristics		% (*N*)
**Gender**	Female	78.3 (731)
	Male	20.7 (194)
	Other	0.9 (8)
	Prefer not to say	0.1 (1)
**Age (years)**	18–24	4.8 (45)
	25–34	12.3 (115)
	35–44	12.4 (116)
	45–54	26.2 (244)
	55–64	27.3 (255)
	65–70	7.9 (74)
	Over 70	9.1 (85)
**Ethnicity**	White	96.6 (901)
	Mixed/multiple ethnic	0.6 (6)
	Asian/Asian British	0.5 (5)
	Black/African/Caribbean/Black British	0.2 (2)
	Chinese	0.4 (4)
	Other ethnicity	0.4 (4)
	Prefer not to say	1.3 (12)
**Companion animal ownership**	Yes	88.7 (828)
**Companion animal species**	Dogs	64.6 (603)
	Cats	37.7 (352)
	Small mammals	9.7 (91)
	Fish	7.8 (73)
	Horses or ponies	5.8 (54)
	Birds	4.8 (45)
	Reptiles	4.2 (39)
	Farm animals	1.8 (17)
	Amphibians	0.9 (8)
**Companion animals with a special role**	Emotional support animals	4.9 (46)
	Therapy animals	3.5 (33)
	Assistance dogs (e.g., guide dogs)	2.0 (19)
	Working dogs	1.0 (9)
**Interaction with non-companion animals**	Feeding/watching birds in my garden	64.2 (600)
	Feeding/watching other wildlife in my garden	35.5 (332)
	Watching wildlife in nature	48.1 (450)
	Volunteering/working for animal rescue organisations or sanctuaries	7.9 (74)
	Riding horses that I am not the main caretaker of	1.8 (17)
	Visiting/watching farm animals close to my home	16.5 (154)
	Following wildlife webcams online	13.6 (127)
	Other enjoyable activities involving animals that you are not the main caretaker of	17.1 (160)
**Cohabitation**	Live alone	24.5 (229)
	With partner/spouse	56.3 (526)
	With children <18 years	18.1 (169)
	With adults 18–70 years old	21.0 (196)
	With adults >70 years	4.9 (46)
	With persons who may be vulnerable to COVID-19	11.3 (106)
**COVID-19 lockdown social isolation status**	Socially isolating	41.2 (385)
	Not socially isolating	58.8 (549)

COVID-19: coronavirus disease 2019.

**Table 2:** Themes and associated sub-themes.

Theme One: Positive impact of animal ownership during the COVID-19 lockdown Amelioration of wellbeing and mental health Reduction of loneliness and the facilitation of social support Encouragement of physical activity Increased appreciation of animals during the COVID-19 lockdown
Theme Two: Negative impact and concerns of animal ownership during the COVID-19 lockdown Concerns over animals carrying the COVID-19-causing virus Animals’ potential separation-related problems and returning to work after the COVID-19 lockdown General animal welfare worries Financial difficulties and concerns
Theme Three: Grief and loss of an animal during the COVID-19 lockdown
Theme four: Positive impact of interactions with a non-companion animal during the COVID-19 lockdown

## Data Availability

The data presented in this study are openly available on the OSF repository via the following URL: https://osf.io/m9846/.
